# Emotion and Interhemispheric Interactions in Binocular Rivalry

**DOI:** 10.1068/ig2

**Published:** 2013-10-01

**Authors:** K L Ritchie, R L Bannerman, A Sahraie

**Affiliations:** University of Aberdeen, United Kingdom; University of Aberdeen, United Kingdom; University of Aberdeen, United Kingdom

## Abstract

Previous research has shown that fear-related stimuli presented in peripheral vision are preferentially processed over stimuli depicting other emotions. Furthermore, emotional content can influence dominance duration in binocular rivalry, with the period of dominance for an emotional image (e.g. a fearful face) being significantly longer than a neutral image (e.g. a neutral face or a house). Experiment 1 of the current study combined these two ideas to investigate the role of emotion in binocular rivalry with face/house pairs viewed in the periphery. The results showed that faces were perceived as more dominant than houses, and fearful faces more so than neutral faces, even when viewed in the periphery. Experiment 2 extended this paradigm to present a rival pair in the periphery in each hemifield, with each eye either viewing the same stimulus in each location (traditional condition), or a different stimulus in each location (Diaz-Caneja condition). The results showed that the two pairs tended to rival in synchrony only in the traditional condition. Taken together, the results show that face dominance and emotion dominance in binocular rivalry persist in the periphery, and that interhemispheric interactions in binocular rivalry depend on an eye- as opposed to an object-based mechanism.

